# A CRISPR/Cas9-based method and primer design tool for seamless genome editing in fission yeast

**DOI:** 10.12688/wellcomeopenres.10038.3

**Published:** 2017-05-05

**Authors:** María Rodríguez-López, Cristina Cotobal, Oscar Fernández-Sánchez, Natalia Borbarán Bravo, Risky Oktriani, Heike Abendroth, Dardan Uka, Mimoza Hoti, Jin Wang, Mikel Zaratiegui, Jürg Bähler

**Affiliations:** 1 Research Department of Genetics, Evolution and Environment, University College London, London, UK; 2 Department of Molecular Biology and Biochemistry, Rutgers University, Piscataway, USA

**Keywords:** S. pombe, CRISPR-Cas9, gene deletion, mutagenesis, genome editing, sgRNA, PCR primer design

## Abstract

In the fission yeast *Schizosaccharomyces pombe *the prevailing approach for gene manipulations is based on homologous recombination of a PCR product that contains genomic target sequences and a selectable marker. The CRISPR/Cas9 system has recently been implemented in fission yeast, which allows for seamless genome editing without integration of a selection marker or leaving any other genomic ‘scars’. The published method involves manual design of the single guide RNA (sgRNA), and digestion of a large plasmid with a problematic restriction enzyme to clone the sgRNA. To increase the efficiency of this approach, we have established and optimized a PCR-based system to clone the sgRNA without restriction enzymes into a plasmid with a dominant *natMX6 *(nourseothricin) **selection marker. We also provide a web-tool, CRISPR4P, to support the design of the sgRNAs and the primers required for the entire process of seamless DNA deletion. Moreover, we report the preparation of G1-synchronized and cryopreserved *S. pombe* cells, which greatly increases the efficiency and speed for transformations, and may also facilitate standard gene manipulations. Applying this optimized CRISPR/Cas9-based approach, we have successfully deleted over 80 different non-coding RNA genes, which are generally lowly expressed, and have inserted 7 point mutations in 4 different genomic regions.

## Introduction

The fission yeast *Schizosaccharomyces pombe* is a potent genetic model organism. Gene deletions and other genetic manipulations in *S. pombe* are most commonly performed in a single-step by transformation of a PCR product, which includes a selectable marker gene along with flanking regions to target the genomic region to be manipulated ^1^. Several techniques have been developed to circumvent complications caused by selectable markers, including the LoxP-Cre recombinase system ^2^, the *rpl42* (cycloheximide resistance)-based method ^3, 4^, the pop-in/pop-out methods ^5^, a CRISPR method based on fluoride resistance ^6^, or a recent method for scar-less gene tagging ^7^. However, these methods have drawbacks that limit their applicability: they either involve two transformations or selection steps, leave ‘DNA-scars’, affect cellular physiology, or can only be used in specific genetic backgrounds.

The recent emergence of the prokaryotic CRISPR/Cas9 system for genome editing now provides the opportunity for efficient gene manipulation without any markers ^8– 10^. Such seamless genome editing offers several advantages: 1) it allows targeting of multiple genetic manipulations to the same strain without restrictions due to markers or any marker recycling; 2) it avoids indirect physiological effects, which accompany some markers ( 11; M. R.-L. and C. C., unpublished observations); and 3) it limits the perturbation of the local chromatin and transcriptional environment to the gene manipulation of interest.

The CRISPR/Cas9 genome editing system has recently been implemented in fission yeast by applying the promoter/leader sequence of K RNA ( *rrk1*) and a hammerhead ribozyme to express the single guide RNA (sgRNA) ^12^. The Cas9 protein acts as an RNA-guided endonuclease that binds to a protospacer adjacent motif (PAM) site and introduces a double-strand break (DSB) three base pairs upstream of the PAM site in the spacer sequence. The cell can then repair this DSB either by non-homologous end joining, which will introduce point mutations or indels, or by efficient homologous recombination if the cell is provided with a suitable template. This approach allows for the precise editing of genomic locations without the need of any selectable marker, since cells that do not repair the DSB will die. Genome editing in *S. pombe* with CRISPR/Cas9 involves the manual identification of unique PAM and spacer sequences (sgRNA) and cloning of these sequences into an expression plasmid with a *Csp*CI restriction site to produce the sgRNA. Overexpression of the Cas9 enzyme is detrimental for *S. pombe* growth, which is partially circumvented by co-expression of the sgRNA and Cas9 from the same plasmid ^12^. However, the resulting large plasmid (~11 kb) is difficult to work with, and the *Csp*CI digestion required for cloning is often very inefficient. Accordingly, we and others have encountered serious problems in implementing the CRISPR/Cas9 system.

Here, we present a PCR-based, rapid and efficient method for the seamless deletion of any DNA sequence in the *S. pombe* genome *,* or other genome manipulations, such as point mutations, by applying modifications and optimizations of the CRISPR/Cas9 system. We also provide the CRISPR4P web tool to design the different types of primers required for deletion of any genomic region: PCR-based sgRNA cloning, PCR-based synthesis of DNA template for deletion by homologous recombination, and checking primers to confirm the deletion. Furthermore, we have modified a protocol for the generation of cryopreserved *S. pombe* cells ^13^, by implementing G1 synchronization and optimizations, which substantially increases the efficiency of successful transformations, especially for regions that are difficult to delete. This protocol may also facilitate the manipulation of genomic regions using the traditional method ^1^.

## Overview of approach

**Figure 1.**
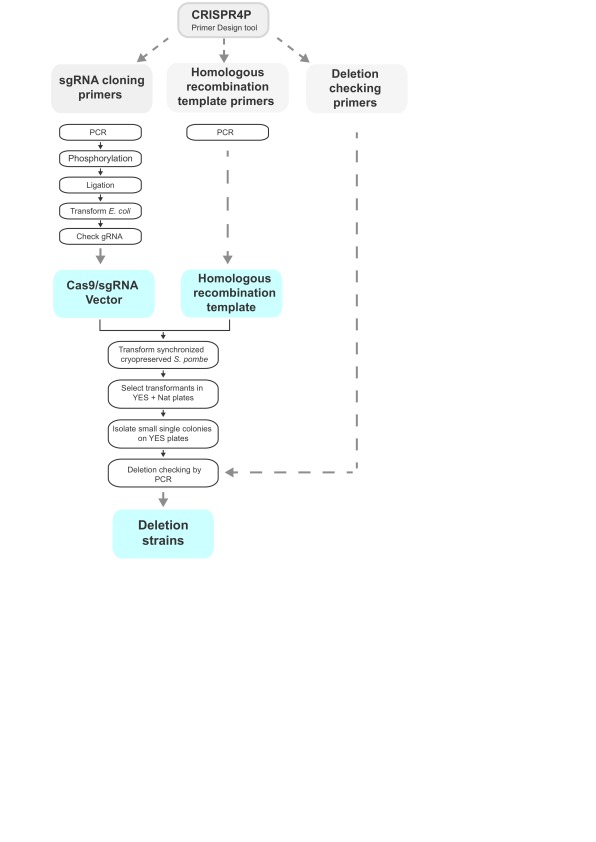
Flow diagram for CRISPR/Cas9-based approach for seamless genome editing in fission yeast. sgRNA, single guide RNA.

The main steps of the CRISPR/Cas9-based method to generate gene deletions are briefly highlighted below. Figure 1 provides a flow diagram of the main steps. A more detailed methodology is available at the end of the manuscript and as a PDF in Supplementary File 1. The entire procedure takes about 8 days, including about 5 days for incubation.

**Figure 2.**
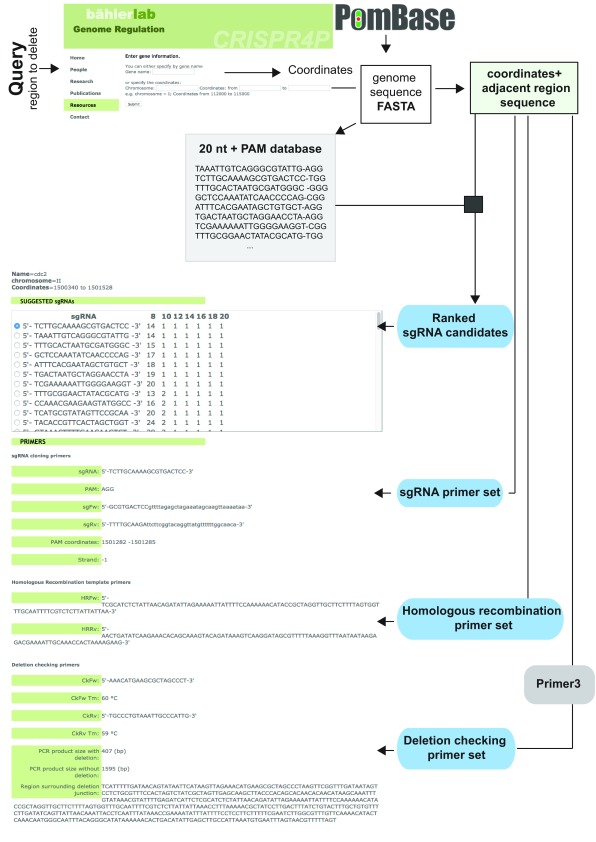
Overview of workflows for CRISPR4P tool for sgRNA and PCR primer design. sgRNA, single guide RNA; PAM, protospacer adjustment motif. In the table ‘Suggested sgRNAs’, the numbers of genomic sgRNA sequences that share a given number of nucleotides are indicated to the right of each sgRNA. See main text for details.

**Figure 3.**
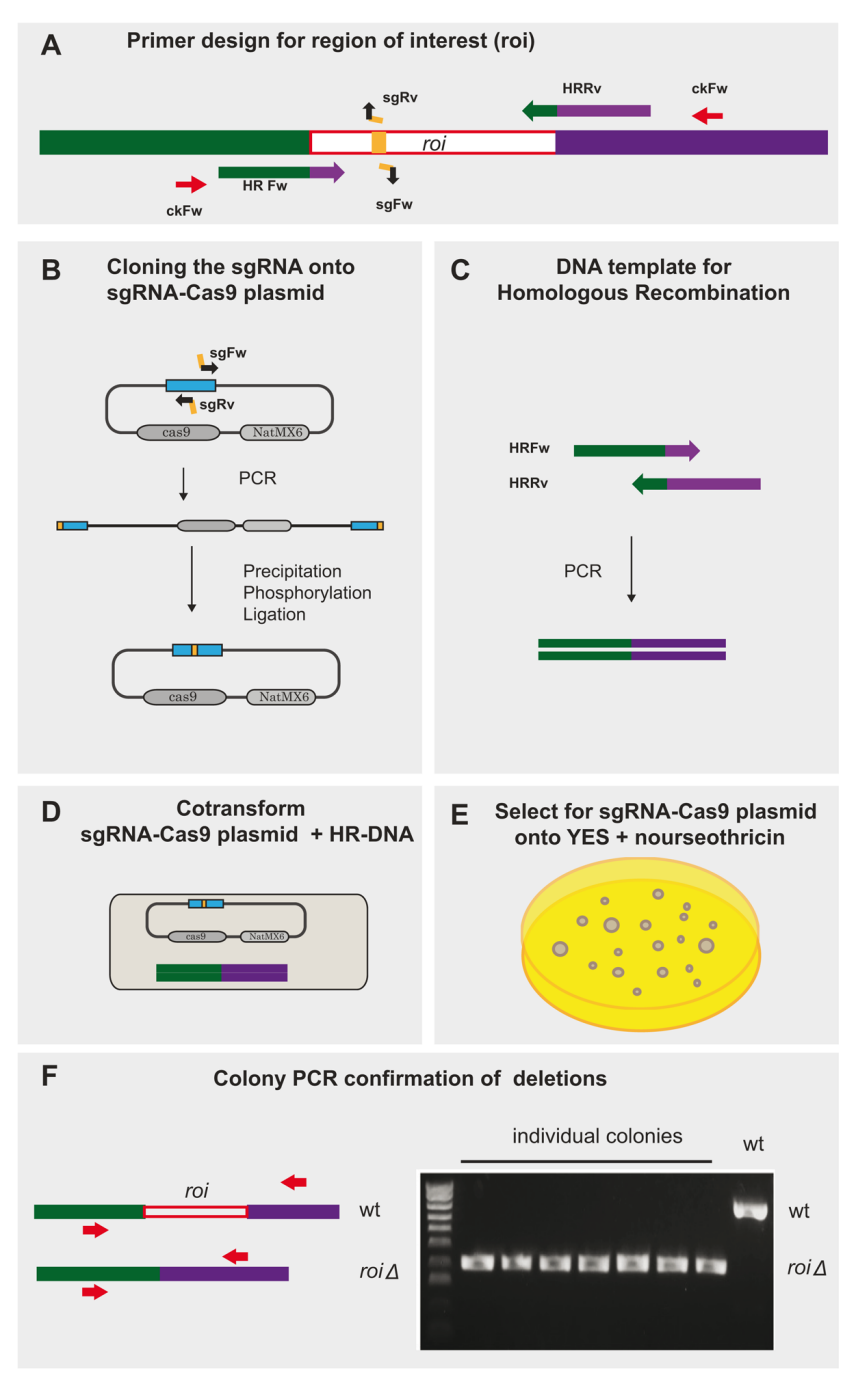
Scheme of key steps for CRISPR/Cas9-based method and primer design. sgRNA, single guide RNA.

**Figure 4.**
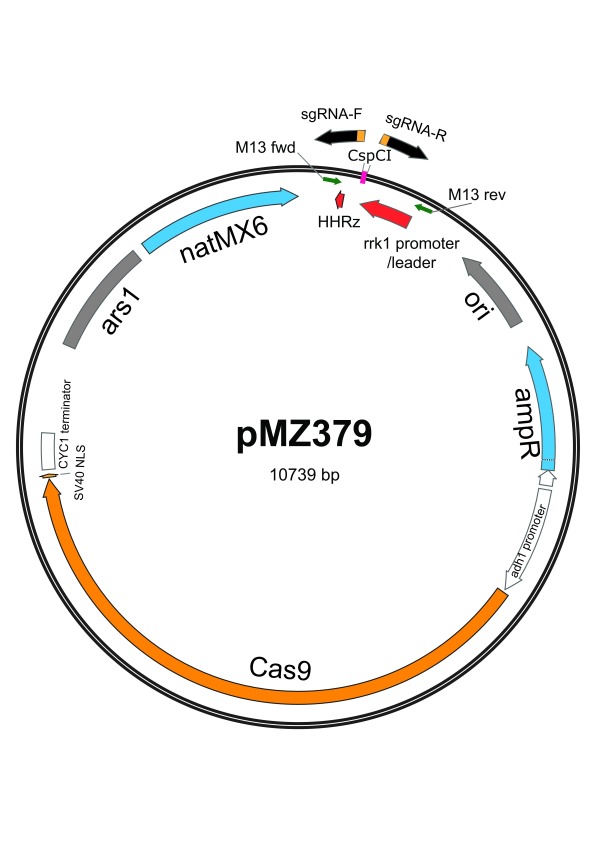
Map of pMZ379 plasmid. The primers to clone sgRNA are indicated by purple arrows. The primers for sequencing insertion of sgRNA are indicated by green arrows. Image adapted from *snapGene* viewer. sgRNA, single guide RNA.

1. Identify suitable sgRNAs to target region of interest using CRISPR4P tool ^14^ ( bahlerlab.info/crispr4p) ( Figure 2).
2. Design of primers required for whole process using CRISPR4P ( Figure 3A): 1) sgRNA cloning; 2) synthesis of DNA template for homologous recombination (HR template) for gene deletion; and 3) checking primers to confirm gene deletion.
3. Clone sgRNAs into nourseothricin-selectable plasmid pMZ379 that contains Cas9 enzyme gene, the *natMX6* selection marker and the *rrk1* promoter/leader ( Figure 3B; Figure 4).
4. Generate HR template by PCR using primers with sequences flanking the region of interest and overlapping at their 3’ ends ( Figure 3C).
5. Delete region of interest by co-transforming sgRNA/Cas9-plasmid and HR template into *S. pombe* cells that have been synchronized and cryopreserved to increase transformation efficiency ( Figure 3D).
6. Select the smallest colonies from selective plate ( Figure 3E) and check these colonies for deletion junction by colony PCR ( Figure 3F).

Note that this approach can be adapted for applications other than gene deletions, such as insertion of point mutations or tags. The CRISPR4P tool allows the user to identify possible sgRNA sequences in any region of interest for other applications of the CRISPR/Cas9-based genome editing.

## CRISPR4P primer design tool

Available primer design programs for gene targeting in *S. pombe* allow the manipulation of coding sequences using the standard PCR-based method ^15^, or rely on current gene annotations to generate a database that contains primers for deletion of non-coding RNAs, 3’-UTRs or tRNAs ^16^. We have designed an online tool, written in Python 2.7 ( www.python.org/), to help with the design of all the different primers required for CRISPR/Cas9-based deletion of virtually any region in the *S. pombe* genome. This tool, named CRISPR4P (CRISPR ‘for’ *Pombe* or CRISPR *Pombe* PCR Primer Program), is freely available from our website ( bahlerlab.info/crispr4p) ^14^. CRISPR4P designs PCR primers for sgRNA cloning and primers to generate the HR template, and also checks primers to verify gene deletions. Figure 2 provides an overview of the workflows for CRISPR4P, and Figure 3A provides an overview of the different primers that can be designed by CRISPR4P.

### Design of sgRNA

The sgRNA targets the Cas9 enzyme to its recognition site to generate a DSB upstream of the PAM sequence. However, it has been reported that Cas9 can also generate DSBs in other genomic sequences that contain a few mismatches compared to the sgRNA ^17, 18^, and even in sites that cannot be predicted simply by sequence homology ^19^. Thus, it is important to choose a suitable target region to maximize the specificity and avoid undesirable off-target effects. Mismatches within the 12 nucleotide ‘seed’ sequence, immediately upstream of the PAM sequence, reduce the nuclease activity of the Cas9 enzyme, and must therefore be avoided for the target sequence ^20^. On the other hand, such mismatches in similar sequences elsewhere in the genome will reduce the likelihood of Cas9 targeting. Multiple tools are becoming available for the prediction of sgRNAs and off-target effects (see 21 for a review), but not all include the *S. pombe* genome, and there have been no studies into the issue of off-target effects in *S. pombe*. CRISPR4P facilitates the design of sgRNAs and provides basic information on the similarity of sgRNA sub-sequences to other genomic sgRNA sequences to minimize off-target effects. In the case of gene deletions, there is considerable flexibility with respect to sgRNA selection because the targeting is not limited to a narrow region.

CRISPR4P has scanned the *S. pombe* genome, downloaded from PomBase, for all possible 3-nucleotide *Streptococcus pyogenes* Cas9 PAM sites (5’-NGG-3’), and stored this information together with the sequences of the 20 nt upstream of all these PAM sites (sgRNA sequences), thus generating a database of all possible genomic sgRNAs plus PAM sites. Users can input their target regions either by gene name or genomic coordinates, with the latter providing the flexibility to delete any region of interest, such as regulatory sequences, non-coding RNAs, or specific sub-regions of genes. If the input is a gene name, the coordinates of the coding sequence are calculated based on PomBase annotation (genome assembly ASM294v2, version 55) ( http://www.pombase.org/) ^22, 23^. CRISPR4P then examines the nucleotide string within the input coordinates of the target sequence for PAM sites along with the upstream 20 nt sgRNAs using the genomic database of all sgRNAs plus PAM sites. CRISPR4P is not an off-target scorer, but helps users in the selection of suitable sgRNAs, based on basic concepts of similarity to other regions. Our premise is that the chosen 20 nt sgRNA should be unique in the genome, and only unique sgRNAs will therefore be provided in the output. In addition, CRISPR4P then presents all the possible sgRNAs in the target region, ranked by similarity to other putative sgRNAs anywhere in the genome. The data to the right of each sgRNA indicates the numbers of genomic sgRNA sequences that share a given number of nucleotides (starting from the 5’ end of the PAM sequence), scanning the sgRNA from 8 bp up to 20 bp every 2bp. To minimize any off-target effects, the 12 nt ‘seed’ sequence immediately upstream of the PAM site should ideally be unique in the genome. Furthermore, we do recommend the use of at least two different sgRNAs for any given deletion construct and to test the phenotypes of several independent deletion strains from each transformation. Any specific off-target mutation is unlikely to occur independently in different clones, and even less likely to occur with different sgRNAs.

A specific sgRNA can be selected by clicking the round button to the left of the sequence; CRISPR4P will then provide the corresponding outputs at the bottom, including the sgRNA sequence together with its coordinates and the two primer sequences required to clone the sgRNA into the plasmid pMZ379 by PCR ( Figure 3A and B). CRISPR4P also provides two other sets of PCR primers described below.

### Primer design for HR template

CRISPR4P selects 80 nt up- and down-stream of the target sequence to be deleted and joins these sequences together into a 160 nt long HR template sequence to target the region of interest for seamless deletion by homologous recombination. This ‘junction’ sequence is then used to design the primers to generate the HR template DNA by PCR amplification ( Figure 3A and C). The forward primer (HRfw) contains the 100 nt from the 5’-end of the HR template, and the reverse primer (HRrv) are the reverse complementary 100 nt from the 3’-end of the HR template. We have found that 20 nt of overlapping region between these two PCR primers are sufficient to generate the HR template.

### Primer design to check deletion junction

CRISPR4P also provides two PCR primers to check the seamless deletion junction. These primers are positioned up- and down-stream of the HR template region. First, CRISPR4P generates *in silico* a region surrounding the deletion junction by joining the 250 nt immediately up- and down-stream of the junction. This sequence is then used as the input for the Python implementation of Primer3 ( http://primer3.ut.ee/) ^24^ to design checking primers ( Figure 3A and F). The output of this third module is the region surrounding the deletion junction (which can be used for verification of the junction by DNA sequencing), the two checking primers with their melting temperatures (Tm), and the expected sizes of the PCR products obtained for either successful deletion or without deletion (wild-type).

## Rationale for optimization of experimental protocols

### Cloning of sgRNA

Since the CspCI digestion of the plasmid containing Cas9 and the *rrk1*-guided sgRNA is often inefficient, it can be very difficult to clone sgRNAs into the plasmid optimized for CRISPR/Cas9 gene editing in *S. pombe*. We therefore devised alternative approaches for the introduction of the sgRNA into the pMZ379 plasmid (available through Addgene; plasmid no., 74215). The new Cas9-sgRNA plasmid pMZ379 contains a dominant selection marker that does not rely on auxotrophy ( Figure 4). This plasmid enables the application of the CRISPR-Cas9 technique in any genetic background. Moreover, we have observed that the use of auxotrophic markers, such as *ura4,* can lead to undesirable physiological side effects (M.R.-L. and J.W., unpublished observations), as also observed for *S. cerevisiae* ^11^.

The first method introduces the sgRNA sequence via the 5’ ends of the primers used for PCR amplification of the pMZ379 plasmid sequence, followed by phosphorylation and ligation of the PCR product to reconstruct a new circular plasmid containing the desired sgRNA. We provide detailed PCR optimizations and other methods to deal with the large (~11kb) pMZ379 plasmid, which are critical for the success of the approach.

We observed that during sgRNA cloning errors sometimes occur with sequences containing microhomology regions next to the junctions. We have therefore developed an alternative, ligation-free method for the PCR-cloning of sgRNAs into the pMZ379 plasmid, using two longer primers that each contain the complete 20 nt sgRNA sequences, in opposite orientation, at their 5’ ends. In this ligation-free method, plasmid recircularization is carried out by the bacteria after transformation. This improved method thus avoids errors occurring during the ligation step that is required for the first method, and is faster and less expensive. CRISPR4P designs primers for both the traditional and ligation-free cloning method.

### G1 synchronization and cryopreserved competent cells

The activity of the prokaryotic Cas9 enzyme is likely increased in sites with more accessible chromatin and lower nucleosome occupancy. In mammalian cells, for example, Cas9 is more effective for sgRNAs that target coding sequences where chromatin is more open compared with other regions ^25^. This could cause problems for deleting or editing poorly transcribed and inaccessible regions, as we have observed for several non-coding RNAs (M.R.-L., C.C. and N.B.B., unpublished data),. Moreover, proliferating *S. pombe* cells spend most of their time in G2 phase with a 2C DNA content. In these cells, two genomic copies need to be successfully modified by CRISPR/Cas9, and if only one copy is modified, the wild-type copy could be used as a template for homologous repair of the DSB. Therefore, it is likely that the efficiency of CRISPR/Cas9 genome editing is increased in G1 cells that contain a 1C of DNA.

**Figure 5.**
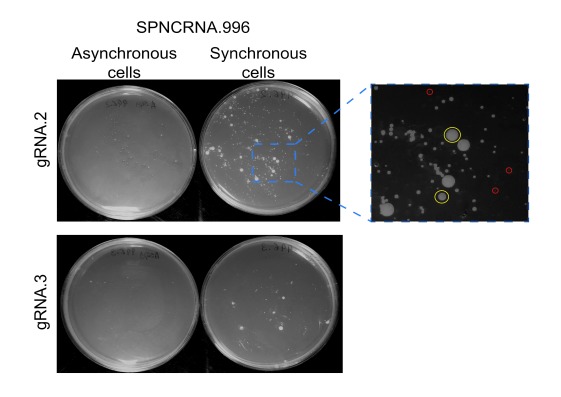
Improved transformation using G1-synchronized, cryopreserved cells. Wild-type cells were cultured in EMM medium and either subjected to nitrogen starvation for 2 hrs (synchronous) or maintained on normal EMM medium (asynchronous) at 25°C. The same number of cells were then made competent and frozen for synchronous and asynchronous cultures. Cryopreserved synchronous and asynchronous cells were transformed with the same amount of DNA for two different sgRNAs, as indicated at top and left. Cells were incubated at 32°C for 4 days, revealing a greatly increased transformation efficiency of the synchronous cells, with no colonies present for unsynchronized cells. Transformation of synchronous cells consistently resulted in 3-fold to over 1000-fold higher numbers of colonies than transformation of asynchronous cells.The enlarged image indicates the smallest colonies that are much more likely to contain successful deletions (red circles) than the large colonies (yellow circles).

Having these issues in mind when encountering low efficiencies for the CRISPR/Cas9 system, we implemented the synchronization of *S. pombe* cells in G1 using a simple nitrogen starvation protocol. This treatment not only greatly increases the proportion of cells with a 1C DNA content, but also substantially remodels the transcriptional programme ^26, 27^, which can render many genomic regions more accessible. Moreover, we optimized a protocol for cryopreservation of competent, G1-synchronized cells ^13^, which greatly improves transformation and deletion efficiencies. Accordingly, we observed dramatically enhanced transformation rates when using G1-synchronized and cryopreserved cells ( Figure 5).

## Application of CRISPR/Cas9-based approach

**Figure 6.**
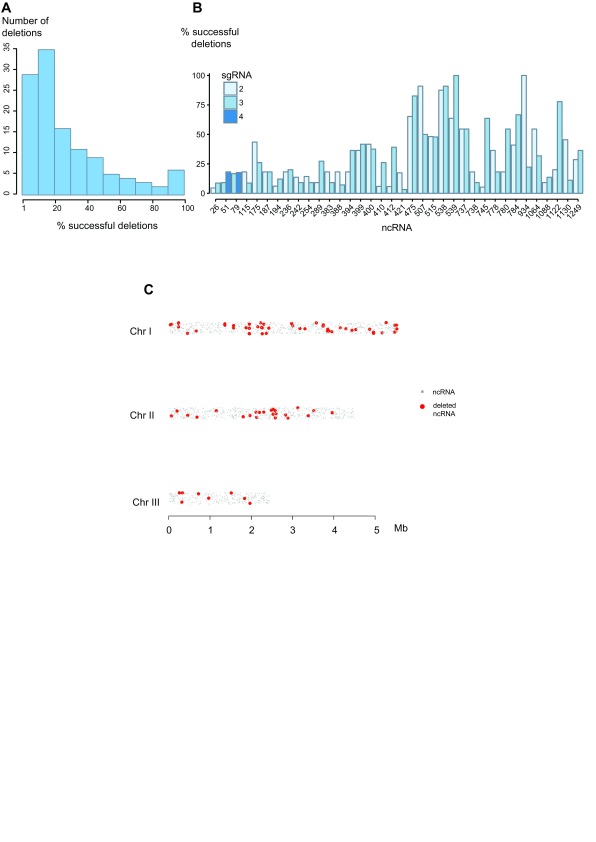
Deleting non-coding RNAs using CRISPR/Cas9-based approach. ( **A**) Percentage of successful deletions among all sgRNAs that yielded at least one successful deletion for different non-coding RNA genes, with data from 120 different deletions aggregated. ( **B**) Data on efficiencies of successful deletions for 36 non-coding RNA genes, for transformation with different sgRNAs (color-coded 2-4). In these cases, sgRNA1 were designed manually and did not yield successful deletions, whereas the sgRNAs 2-4 designed by CRISPR4P proved to be largely successful, and sgRNA4 were used for the two genes where sgRNA2 did not work. Note the greatly varying success rate for different genomic loci and for different sgRNAs.The data used for the graphs in ( **A**) and ( **B**) are provided in Supplementary Table 2. ( **C**) Genomic locations of all annotated non-coding RNA genes (small grey dots) and the non-coding RNA genes which we have deleted (red dots).

Using this optimized approach, we have deleted over 80 non-coding RNA genes 36 of which were deleted using 2 different sgRNAs (all primers for the deletions can be found in Supplementary Table 1). The efficiencies for successful deletions vary considerably for different genes ( Figure 6A) and for different sgRNAs targeting the same gene ( Figure 6B), with success rates ranging from 3% to 100%. For example, for the deletion of SPNCRNA.745, we obtained 5% positive colonies with one sgRNA (sgRNA.745.2) and 64% positive colonies with another one (sgRNA.745.3). Thus, using at least 2 different sgRNAs per deletion not only minimizes the risk of being misled by phenotypes from off-target effects but it also maximizes the chance of successful deletion. The deleted non-coding RNA genes were spread across all 3 chromosomes ( Figure 6C).

**Figure 7.**
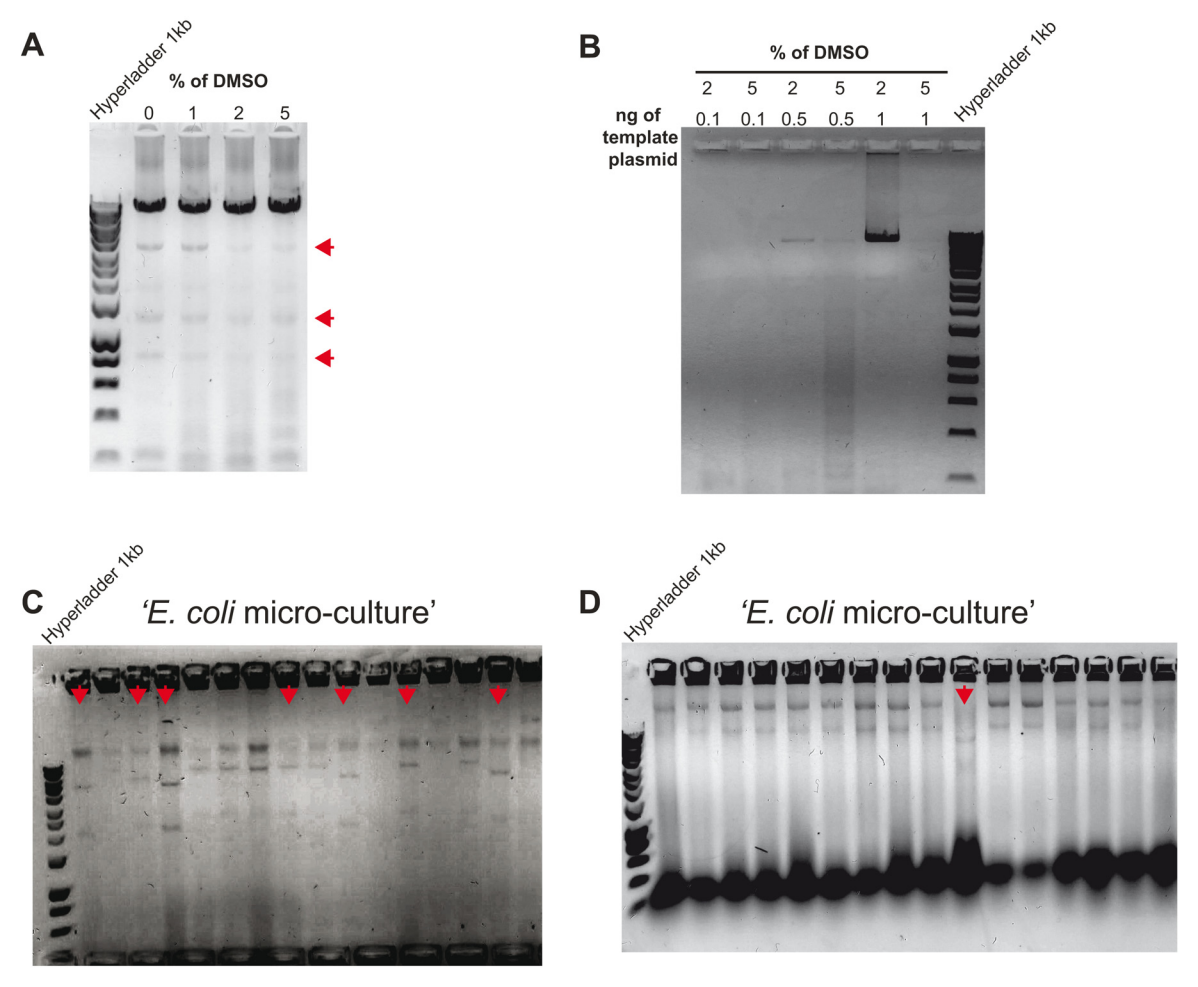
PCR cloning of sgRNA into pMZ379 plasmid and ‘ *E. coli* micro cultures’. ( **A**) Gel of non-optimized PCR reactions for sgRNA cloning. Red arrows indicate shorter, unspecific products that need to be removed to avoid faulty plasmids. ( **B**) As in (A), but with optimized PCR reaction which eliminates unspecific products (see protocol for details). ( **C**) Gel of sgRNA-pMZ379 plasmid using non-optimized PCR reactions for sgRNA cloning. Each well contains DNA from different colony of same transformation. Red arrows indicate faulty cloning reactions containing plasmids of different sizes. ( **D**) As in (C), but with optimized PCR reaction used for cloning which largely prevents faulty cloning reactions.

We have also successfully and efficiently introduced 7 point mutations in 4 different genomic regions using the CRISPR/Cas9 system, without leaving any other scars in the genome. Sites can only be mutated if they are located within the first 8–10 nt upstream of the PAM site. If no PAM site is available within this distance, a workaround could be applied by inserting two mutations as follows: use the HR template to introduce a synonymous change 8–10 nt upstream of the nearest PAM site, to prevent re-cutting by Cas9, plus the desired mutation where required. Mutagenesis is a particularly attractive application of the CRISPR/Cas9 method. In principle, the CRISPR/Cas9 system could also be adapted to tag genes, but we have not yet tried this out.

The CRISPR4P tool greatly facilitates the design of the sgRNAs and the different sets of primers required for the entire approach. The sgRNAs designed by CRISPR4P generally showed much higher success rates than manually designed sgRNAs. The current version of CRISPR4P only provides primers for deletion of genomic regions using the CRISPR/Cas9 system. However, CRISPR4P can be used to design sgRNAs to generate point mutations by inputting the coordinates of the region of interest. In future work, we are assembling a database with all sgRNAs used, whether they worked or not, to help with learning the principles for successful sgRNAs in *S. pombe*.

Help box 1. **How to use the CRISPR4P tool.**

- Input region to be deleted, either by Gene name (e.g., *cdc2* or *SPNCRNA.01*) or by Coordinates (e.g., Chromosome *:* *II*; Coordinates: from *1500340* to *1501528*).
- Output list of sgRNAs is ordered by similarity to other genomic sgRNAs, with most unique sgRNA on top; select sgRNA by clicking on radial button at left.
- Output provides 3 types of primers
  - Primers for sgRNA cloning depending on selected sgRNA.
  - Primers to generate HR template.
  - Primers for checking the deletion construct, along with melting temperatures and expected sizes (in nt) of PCR products for successful deletion or without deletion, and along with sequence surrounding the deletion junction.

Help box 2. **Important steps for successful application of CRISPR/Cas9.**

- Select at least 2 sgRNAs per construct with low similarity to other genomic regions.
- The large pMZ379 plasmid is unstable: aliquot and store at -80°C (do not thaw and re-freeze aliquots).
- To avoid generation of partial plasmids during PCR reaction, optimized PCR conditions are required: *Phusion High Fidelity* mastermix, 60°C annealing temperature, 2% DMSO, 1 ng (40 fg/µl) of pMZ379 template, 25 PCR cycles.
- Use high-fidelity polymerase to amplify plasmid and HR templates.
- After bacterial transformation of sgRNA plasmid, perform ‘ *E. coli* micro-cultures’ to reduce number of minipreps.
- Check for correct sgRNA by sequencing Cas9-sgRNA plasmid with M13F primer.
- Synchronize *S. pombe* cells for 2 hrs in EMM without nitrogen before making them competent to increase efficiency of transformation, reduce incubation times, and facilitate deletion of difficult genomic regions.
- To support homologous recombination of HR template, incubate cells for 16 hours in EMM without nitrogen after transformation which avoids need for first plating cells onto YES before replica-plating on selective media (common practice for antibiotic markers selection) and thus prevents cells from proliferating.
- Select smallest colonies from transformation as these are most likely to contain correct deletions. Positive colonies typically appear only 4 days or later after plating, while colonies growing faster are typically negative for the deletion.

## Methods

### Reagents and equipment

▪ pMZ379 plasmid (Addgene, plasmid # 74215)
▪ Phusion® High-Fidelity PCR Master Mix with HF Buffer (NEB, cat. no. M0531S)
   
*CAUTION: this product contains DMSO which is known to be harmful for aquatic life, discard appropriately.*
▪ TopTaq Polymerase (QIAGEN, cat. no. 200201)
▪ dNTPmix 10 µM (Bioline, cat. no. BIO-39044)
▪ 5× DNA Loading Buffer Blue (Bioline, BIO-37045)
▪ ExoSAP-IT PCR Product Cleanup (Affymetrix, cat. no. 78200 200 UL)
   
*CAUTION: this product is irritant and can cause severe eye damage, wear protective eye wear.*
▪ Sodium acetate 3M (Thermo Fisher Scientific, cat. no. AM9740)
▪ Ethanol (MERCK, cat. no. 1.08543.0250)
▪ T4 Polynucleotide Kinase (NEB, cat. no. M0201S)
▪ T4 DNA ligase (NEB cat. no. M0202S)
▪ Mix & Go Competent Cells - Strain DH5α, 96 × 50 μl (Cambridge Bioscience, cat. no. T3009
▪ QIAprep Spin Miniprep Kit (QIAGEN, cat. no. 27104)
   
*CAUTION: Buffers P2 and N3 are corrosive and causes skin and eye damage/irritation, wear eye protection and gloves. Buffer PB is highly flammable and causes skin and eye irritation, and possibly dizziness. Keep away from fire, wear protective clothing and eye wear. Avoid inhalation of RNase A.*
▪ Herring sperm DNA 10 µg/µl (Promega, cat. no. D1811)
▪ Lithium Acetate dihydrate (Sigma-Aldrich, cat. no. L-4158-250g)
▪ Gycerol (Fisher Scientific, cat. no. BP229-1)
   
*CAUTION: irritant, wear protective eye wear gloves and clothing.*
▪ Polyethylene glycol 4000 (PEG4000) (VWR cat. no. 26606.293)
▪ Nourseothricin-dihydrogen sulfate (Werner BioAgents cat. no. 5.002.000)
   
*CAUTION: this product is harmful if swallowed, wear protective clothing and eye wear.*
▪ LB Broth Base w/o Trace elements (Formedium, cat. no. AIMLB0110)
▪ Agar powder (VWR, cat. no.20767.298)
▪ Ampicillin (Sigma, cat. no.A9518-25g)
   
*CAUTION: might cause skin irritation and respiratory problems, wear protective eye wear, gloves and avoid inhalation.*
▪ YES BROTH (Formedium, cat. no.PMCUCL1000)
▪ EMM BROTH W/O NITROGEN (Formedium, cat. no. PMD1310)
▪ NH _4_Cl (Sigma, cat. no. 09718/1kg)
   
*CAUTION: NH _4_Cl is toxic if swallowed, can cause eye irritation, wear protective clothing and eye wear.*
▪ Ultrapure Agarose (THERMO FISHER, cat. no. 16500500)
▪ 10X Ultrapure TBE buffer (Life tech, cat. no. 15581044)
   
*CAUTION: can cause damage in the unborn child, may cause fertility problems, wear protective clothing and eye wear.*
▪ HyperLadder 1kb (Bioline, cat. no. BIO-33053)
▪ Freeze 'N Squeeze™ DNA Gel Extraction Spin Columns (Bio-Rad, cat. no. 7326166)
▪ Primer M13F: tgtaaaacgacggccagt

**Step-by-step procedure** (can be downloaded as Supplemental File 1)

*Selection of sgRNAs and primers to delete region of interest*

1. Use CRISPR4P ( bahlerlab.info/crispr4p) to input desired deletion target by gene name or by coordinates as chromosome (in roman numeral), start and end sites. Select sgRNA by clicking button at left to display primers required for this sgRNA. We have found that there is no need for HPLC-purified oligos, desalted oligonucleotides synthetized by our usual provider (Life Technologies) work well for the entire procedure, substantially reducing the cost of the deletions.

CRITICAL STEP: CRISPR4P allows selection from all unique sgRNAs present within the input target region. The sgRNAs are ranked from least likely to most likely to have off-target effects, based on similarity of sub-sequences to other genomic sgRNAs. It is recommended to choose at least two sgRNAs from the top of the list.

*Cloning sgRNA into pMZ379 plasmid (TIME: ~9 hours)*

2. Prepare master mix for PCR to clone sgRNA into pMZ379 plasmid as in table below.

Use sgRNA cloning primers designed by CRISPR4P

**Table:** 

	Final concentration	Volume per reaction (25 µl)
pMZ379 DNA (1 ng/µl) Primer mix (10 µM per primer) Phusion HF-buffer (2X) DMSO (100%) H _2_O	1 ng (40 fg/µl) 0.4 nM/primer 1× 2%	1 µl 1 µl 12.5 µl 0.5 µl 10 µl

CRITICAL STEP: pMZ379 is unstable and should be stored in 1 ng/µl aliquots at -80°C, discard after thawing.

3. Perform PCR following protocol below.

**Table:** 

Number of cycles	Temperature	Duration
1 25 1	98°C 98°C 60°C 72°C 72°C	2 min 10 sec 30 sec 5 min 30 sec 5 min

CRITICAL STEP: PCR conditions have been adjusted for Phusion High-Fidelity Polymerase to minimize number of unspecific PCR bands Figure 7A, B.

4. Check PCR products by running 5 µl on 0.7% agarose TBE gel ( Figure 7A).

**NOTE**: For the ligation-free method, go to *Transformation of chemically competent E. coli cells* (Step 19), and transform cells directly with 5µl of PCR product.

5. **Optional step:** Add 8 µl of ExoSAP-IT PCR Product Cleanup to 20 µl of PCR reaction. Incubate in PCR machine for 15 min at 37°C and for 15 min at 80°C. 

6. Precipitate DNA by adding 60 µl of 100% ethanol and 6 µl of 3M sodium acetate.

7. Incubate 30 min at -20°C.

8. Centrifuge for 20 min at 20,000g, 4°C to precipitate PCR product.

9. Remove supernatant.

10. Add 50 µl of 70 % ethanol (do **not** resuspend pellet).

11. Centrifuge for 10 min at 20,000g, 4°C.

12. Remove supernatant and air dry pellet.

13. Resuspend pellet in 20 µl of H _2_0.

PAUSE POINT: PCR product can be stored at -20°C until further processing.

14. Phosphorylate the 5’ ends of PCR product by preparing master mix as below.

**Table:** 

	µl per reaction in 30 µl final volume
PCR product T4 DNA ligase buffer T4 PNK H _2_O	20 3 1 6

CRITICAL STEP: DNA ligase buffer is used because it provides the ATP required for the phosphorylation reaction (as recommended by manufacturer), and this enzyme exerts 100% activity in this buffer.

15. Incubate 30 min at 37°C.

16. Inactivate the enzyme by incubating for 20 min at 65°C.

PAUSE POINT: Phosphorylated DNA can be stored at -20°C until further processing.

17. Ligate plasmid ends by preparing master mix below

**Table:** 

	µl per reaction in 10 µl final volume
Phosphorylated DNA T4 DNA ligase buffer T4 DNA ligase	8 1 1

18. Ligate for 16 hrs at 16°C.

PAUSE POINT: Ligated DNA can be stored at -20°C until further processing.

*Transformation of chemically competent E. coli cells (TIME: 30 min)*

Cambridge Bioscience Mix & Go Competent Cells can be transformed using a short protocol provided by manufacturer.

19. Prepare LB-agar plates containing 75 µg/ml of ampicillin, and incubate them at 37°C for 15 min.

20. Thaw one aliquot (per transformation) of Mix & Go Competent Cells (DH5α strain) on ice.

21. Add 5 µl of ligated plasmid to cells, mix gently by tapping with finger.

CRITICAL STEP: Do not pipette competent cells.

22. Incubate cells on ice for 5 min.

23. Plate whole mixture of cells and DNA onto pre-warmed (37°C) LB-ampicillin plates.

24. Incubate plates for 20 hrs at 37°C.

*Confirmation of sgRNA cloning (TIME: ~2 days)*

Smaller, unspecific products during PCR amplification can lead to cloning mistakes ( Figure 7A). But even in the absence of such unspecific PCR products, the plasmid can recombine during cloning, which results in aberrant sizes. To confirm that the sgRNA has been cloned correctly and that there are no mutations or rearrangements, we recommend to test about 12 colonies for each transformation by performing an ‘ *E. coli* micro-cultures’, as follows.

25. Prepare ‘ *E. coli* micro-culture’ plate by adding 30 µl of LB + 75 µg/ml of ampicillin onto each well of a sterile 96-well plate.

26. Inoculate different transformant in each well.

27. Close plate with adhesive seal.

28. Incubate cells for 20 hrs at 37°C.

29. Prepare new 96-well plate with 30 µl of LB + 75 µg/ml of ampicillin on each well.

30. Make replica of bacterial micro-culture plate onto the new plate by inoculating 5 µl of original micro-culture into new plate, seal and incubate it at 37°C.

31. Boil original bacterial micro-culture plate for 10 min at 98°C in PCR machine.

32. Let it rest for 2 min at 4°C.

33. Mix with appropriate volume of loading buffer for each well (e.g. 5 µl of 5× loading buffer).

34. Run 20 µl of bacteria-loading buffer mix on 0.7% agarose TBE gel to check for appropriate plasmid size.

CRITICAL STEP: This step will allow to identify plasmids of the wrong size, so that only plasmids of the correct size are selected to test by sequencing ( Figure 7C, D).

35. Prepare 5 ml inoculums of bacteria containing clones of correct size on LB + 75 µg/ml of ampicillin. Cells are taken from replica plate of ‘ *E. coli* micro-culture’. We normally check 1-2 transformants by sequencing.

36. Incubate for 20 hrs at 37°C.

37. Prepare glycerol stock of bacteria by mixing 500 µl of bacterial culture with 500 µl of 50% sterile glycerol, and store at -20°C.

38. With remaining 4.5 ml of bacterial culture perform a ‘mini prep’ with QIAprep Spin Miniprep Kit.

39. Quantify plasmid DNA and send for Sanger sequencing to confirm that correct sgRNA has been cloned. Use primer M13F (TGTAAAACGACGGCCAGT) for sequencing.

PAUSE POINT: Plasmids and glycerol stocks can be stored at -20°C until sequence has been confirmed.

CRITICAL STEP: In case of ligation-mediated sgRNA cloning, the last base pair next to the junction point is deleted in rare cases, leading to 19 nt sgRNA, so it is advisable to check 1–2 colonies for each sgRNA clone to ensure the appropriate sequence of the sgRNA.

*Generation of HR template (TIME: ~ 2.5 hrs)*

40. Prepare PCR mastermix as in table below.

**Table:** 

	final concentration	Volume to add for 1 reaction (50 µl)
HR primer mix (10 µM per primer) Phusion HF-buffer (2X) H _2_O	0.4 nM/primer 1×	2 µl 25 µl 23 µl

41. Perform PCR following protocol below.

**Table:** 

Cycles	Temperature	Duration
1 cycle 30 cycles 1 cycle	98°C 98°C 55°C 72°C 72°C	2 min 10 sec 10 sec 30 sec 5 min

42. Check PCR products by running 5 µl on 1.5 % agarose TBE gel.

PAUSE POINT: HR template can be stored at -20 °C.

*Preparation of synchronized competent cryopreserved S. pombe cells (TIME: ~1.5 days)*

This protocol is a modification of a previously described method ^13^ to prepare 200 ml of competent cells that allows for 40 transformations.

43. Prepare 20 ml preculture in EMM and grow cells by shaking at 32°C for 8–16 hrs.

44. Dilute cells in 200 ml EMM and grow cells until they reach mid-exponential phase (~2 x 10 ^9^ cells in total).

45. Centrifuge cells for 3 min at 1800g, room temperature.

46. Remove supernatant.

47. Wash in one volume (200 ml) of EMM without nitrogen (EMM-N).

48. Repeat steps 45 and 46 once more.

49. Resuspend cells in 200 ml of EMM-N and transfer to sterile flask.

50. Incubate for 2 hrs at 25°C with shaking.

51. Check that cells have become smaller and rounder, under light microscope.

52. Place cell culture on ice for 15 min.

CRITICAL STEP: To maintain integrity and competency of cells, they must be kept at 4°C from this moment.

53. Centrifuge cells for 5 min at 1600g, 4°C and remove supernatant.

54. Resuspend cells on **ice-cold**, sterile water.

55. Centrifuge for 5 min at 1600g, 4°C and remove supernatant.

56. Repeat steps 54 and 55 twice more.

57. Resuspend cells in 2 ml of **ice-cold,** filter-sterilized 30%Glycerol, 0.1M Lithium acetate (pH 4.9), which gives 10 ^9^ cells/ml.

58. Prepare 50 µl cell aliquots in 1.5 ml sterile Eppendorf tubes, place aliquots on ice for 2 min. Each aliquot is for one transformation.

59. Store aliquots at -80°C immediately.

PAUSE POINT: Cryopreserved cells can be stored at -80°C for at least 2 months.

*Transformation of cryopreserved S. pombe cells (TIME: 30 min)*

60. Thaw aliquots of synchronized, cryopreserved cells in dry block at 40°C for 2 min.

61. For each transformation, add 2µl of 10 µg/µl denaturated herring sperm DNA, 10 µl of HR template, and 2 µg of gRNA plasmid (~10 µl of standard mini-prep yield of 200ng/µl).

62. Add 145 µl of 50% PEG4000, mix well and immediately incubate mix for 15 min at 43°C.

63. Centrifuge cells for 3 min at 1600g, room temperature.

64. Remove supernatant and resuspend cells in 1 ml of EMM-N.

CRITICAL STEP: In the case of using auxotrophic mutant, we recommend the addition of 1/10 of usual concentration of relevant supplement. If using an *h ^90^* strain, use EMM with nitrogen to prevent sporulation.

65. Incubate at room temperature for 16 hours, without shaking.

66. Centrifuge cells for 3 min 1600g, room temperature.

67. Remove supernatant and plate all cells on YES plates containing 100 µg/ml of Noursethricin.

68. Incubate plates at 32°C for at least 4 days. In case not getting successful deletions, longer incubations are sometimes required to allow small colonies to grow up.

69. Re-streak smallest colonies onto YES plates. Cas9 expression is deleterious for cells, and re-streaking onto non-selective YES allows for elimination of the Cas9 plasmid.

CRITICAL STEP: It is important to select the smallest colonies present ( Figure 5): Large colonies are likely to emerge from transformants with mutations or rearrangements of Cas9 ^12^, and this problem is compounded in the large and unstable Cas9-sgRNA plasmid. Unpublished data suggest that the mutations happen during *E. coli* growth (large colony counts fluctuate between miniprep cultures but are quite stable within one culture), and may get worse with freeze-thaws of the plasmid (more negative large colonies in freeze-thawed plasmids).

*Checking of deletions by colony PCR (TIME: 4 hrs)*

70. Prepare master mix for PCR reactions following table below:

**Table:** 

Components	Final concentration	Volume per reaction (25 µl total)
Cell colony TopTaq PCR Buffer 10× MgCl _2_ 25 mM Primer mix (10 µM per primer) dNTPs mix (10 µmol per dNTP) Q Solution 5× H _2_O TopTaq DNA Polymerase (5 U/ µl)	-- 1× 0.5 mM 0.4 nM/ each primer 0.4 nmol/ each 1× -	Scoop cells with tip of pipette 2.5 µl 0.5 µl 1 µl 1 µl 5 µl 14.75 µl 0.25 µl

71. For each colony take a little biomass with 10 µl pipette tip and resuspend in PCR mix (with this polymerase there is no need to boil cells prior to PCR).

72. Perform PCR following protocol below.

**Table:** 

Cycles	Temperature	Duration
1 cycle 35 cycles 1 cycle	94°C 94°C 52°C 72°C 72°C	2 min 30 sec 30 sec 2min 30 sec 7 min

73. Add 5 µl of loading dye and load 10 µl of PCR product with loading dye mix on 0.7% agarose gel. The sizes of expected products are indicated in the output of CRISPR4P. (For successful deletions, this will be ~200 bp and for wild-type, the size of target region to delete plus ~200 bp of flanking regions).

OPTIONAL: add 0.5 µl of 10 mg/ml RNAse A solution to mix before loading to remove RNA that might complicate band visualization on gel.

To confirm the deletion junction, the PCR products can be sent for Sanger sequencing (the expected sequence surrounding the deletion junction is provided by CRISPR4P). Figure 3F provides an example of an agarose gel showing successful deletions.

## Data and software availability

CRISPR4P software available from: bahlerlab.info/crispr4p

Latest source code: https://github.com/Bahler-Lab/crispr4p

Archived source code: DOI: 10.5281/zenodo.164683 ^14^

License: MIT

Raw data are deposited in OSF ( https://osf.io/5de22/) DOI: 10.17605/OSF.IO/5DE22 ^28^

## Supplementary material

**Supplementary Table 1:** Single guide RNA sequences and all primers used.

**Supplementary File 1:** Step-by-step protocol.

**Supplementary Table 2:** Contains the data used for the graphs in Figure 6A and B.
