# Supplementary material for: A CRISPR/Cas9-based method and primer design tool for seamless genome editing in fission yeast
Source: Wellcome Open Res. 2017 May 5;1:19. Originally published 2016 Nov 23. [Version 3] doi: 10.12688/wellcomeopenres.10038.3 (PMC5445975; doi:10.12688/wellcomeopenres.10038.3)
Supplement: Supplementary file 2 [file wellcomeopenres-1-12322-s0001.tgz › 565dd348-c005-48b8-a1a4-3e0dd10699d7.docx]

*Selection of sgRNAs and primers to delete region of interest*

1. Use CRISPR4P (<http://bahlerlab.info/crispr4p>) to input desired deletion target by gene name or coordinates as chromosome (in roman numeral), start and end.

**CRITICAL STEP:** CRISPR4P allows selection from all the unique sgRNAs present within the input target region. The sgRNAs are ranked from least likely to most likely to have off-target effects, based on similarity of sequence to other genomic regions. It is recommended to choose at least two sgRNAs on top of list. Select sgRNA by clicking button at left to display primers required for this sgRNA.

We have found that there is no need for HPLC-purified oligos, desalted oligonucleotides synthetized by our usual provider (Life technologies) work well for the entire procedure, substantially reducing the cost of the deletions.

*Cloning sgRNA into pMZ379 plasmid (TIME: ~9 hours)*

1. Prepare master mix for PCR to clone sgRNA into pMZ379 plasmid as in table below.

Use sgRNA cloning primers designed by CRISPR4P

|  | **Final concentration** | **Volume per reaction (25 μl)** |
| --- | --- | --- |
| pMZ379 DNA (1 ng/µl) | 1 ng (40 fg/µl) | 1 µl |
| Primer mix (10 µM per primer) | 0.4 nM/ primer | 1 µl |
| Phusion HF-buffer (2X) | 1x | 12.5 µl |
| DMSO (100%) | 2% | 0.5 µl |
| H_2_O |  | 10 µl |

**CRITICAL STEP:** pMZ379 is unstable and should be stored in 1 ng/µl aliquots at -80**º**C, discard after thawing.

1. Perform PCR following protocol below.

| **Number of cycles** | **Temperature** | **Duration** |
| --- | --- | --- |
| 1 | 98ºC | 2 min |
| 25 | 98ºC | 10 sec |
|  | 60ºC | 30 sec |
|  | 72ºC | 5 min 30 sec |
| 1 | 72ºC | 5 min |

**CRITICAL STEP:** PCR conditions have been adjusted for Phusion High-Fidelity Polymerase to minimize number of unspecific PCR bands (Figure 7A,B).

1. Check PCR products by running 5 µl on 0.7% agarose TBE gel (Figure 7A).

**NOTE:** For the ligation-free method, go to *Transformation of chemically competent E. coli cells* (Step 19), and transform cells directly with 5µl of PCR product.

# Optional step: Add 8 µl of ExoSAP-IT PCR Product Cleanup to 20 µl of PCR reaction. Incubate in PCR machine for 15 min at 37ºC and for 15 min at 80ºC.

1. Precipitate DNA by adding 60 µl of 100% ethanol and 6 µl of 3M sodium acetate.
2. Incubate 30 min at -20**º**C.
3. Centrifuge for 20 min at 20,000g, 4**º**C to precipitate PCR product.
4. Remove supernatant.
5. Add 50 µl of 70 % ethanol (do **not** resuspend pellet).
6. Centrifuge for 10 min at 20,000g, 4**º**C.
7. Remove supernatant and air dry pellet.
8. Resuspend pellet in 20 µl of H_2_0.

**PAUSE POINT:** PCR product can be stored at -20**º**C until further processing.

1. Phosphorylate the 5’ ends of PCR product by preparing master mix as below.

|  | **µl per reaction in 30 µl final volume** |
| --- | --- |
| PCR product | 20 |
| T4 DNA ligase buffer | 3 |
| T4 PNK | 1 |
| H_2_O | 6 |

**CRITICAL STEP:** DNA ligase buffer is used because it provides the ATP required for the phosphorylation reaction (as recommended by manufacturer), and this enzyme exerts 100% activity in this buffer.

1. Incubate 30 min at 37**º**C.
2. Inactivate the enzyme by incubating for 20 min at 65**º**C.

**PAUSE POINT:** Phosphorylated DNA can be stored at -20**º**C until further processing.

1. Ligate plasmid ends by preparing master mix below

|  | **µl per reaction in 10 µl final volume** |
| --- | --- |
| Phosphorylated DNA | 8 |
| T4 DNA ligase buffer | 1 |
| T4 DNA ligase | 1 |

1. Ligate for 16 hrs at 16**º**C.

**PAUSE POINT:** Ligated DNA can be stored at -20**º**C until further processing.

*Transformation of chemically competent E. coli cells (TIME: 30 min)*

Cambridge Bioscience Mix & Go Competent Cells can be transformed using a short protocol provided by manufacturer.

1. Prepare LB-agar plates containing 75 µg/ml of ampicillin, and incubate them at 37**º**C for 15 min.
2. Thaw one aliquot (per transformation) of Mix & Go Competent Cells (DH5α strain) on ice.
3. Add 5 µl of ligated plasmid to cells, mix gently by tapping with finger.

**CRITICAL STEP:** Do not pipette competent cells.

1. Incubate cells on ice for 5 min.
2. Plate whole mixture of cells and DNA onto pre-warmed (37**º**C) LB-ampicillin plates.
3. Incubate plates for 20 hrs at 37**º**C.

*Confirmation of sgRNA cloning (TIME: ~2 days)*

Smaller, unspecific products during PCR amplification can lead to cloning mistakes (Figure 7A). But even in the absence of such unspecific PCR products, the plasmid can recombine during cloning, which results in aberrant sizes. To confirm that the sgRNA has been cloned correctly and that there are no mutations or rearrangements, we recommend to test about 12 colonies for each transformation by performing an ‘*E. coli* micro-culture’, as follows.

1. Prepare ‘*E. coli* micro-culture’ plate by adding 30 µl of LB + 75 µg/ml of ampicillin onto each well of a sterile 96-well plate.
2. Inoculate different transformant in each well.
3. Close plate with adhesive seal.
4. Incubate cells for 20 hrs at 37**º**C.
5. Prepare new 96-well plate with 30 µl of LB + 75 µg/ml of ampicillin on each well.
6. Make replica of bacterial micro-culture plate onto the new plate by inoculating 5 µl of original micro-culture into new plate, seal and incubate it at 37**º**C.
7. Boil original bacterial micro-culture plate for 10 min at 98**º**C in PCR machine.
8. Let it rest for 2 min at 4**º**C.
9. Mix with appropriate volume of loading buffer for each well (e.g. 5 µl of 5x loading buffer).
10. Run 20 µl of bacteria-loading buffer mix on 0.7% agarose TBE gel to check for appropriate plasmid size.

**CRITICAL STEP:** This step will allow to identify plasmids of the wrong size, so that only plasmids of the correct size are selected to test by sequencing (Figure 7C,D).

1. Prepare 5 ml inoculums of bacteria containing clones of correct size on LB + 75 µg/ml of ampicillin. Cells are taken from replica plate of ‘*E. coli* micro-culture’. We normally check 1-2 transformants by sequencing.
2. Incubate for 20 hrs at 37**º**C.
3. Prepare glycerol stock of bacteria by mixing 500 µl of bacterial culture with 500 µl of 50% sterile glycerol, and store at -20**º**C.
4. With remaining 4.5 ml of bacterial culture perform a ‘mini prep’ with QIAprep Spin Miniprep Kit.
5. Quantify plasmid DNA and send for Sanger sequencing to confirm that correct sgRNA has been cloned. Use primer M13F (TGTAAAACGACGGCCAGT) for sequencing.

**PAUSE POINT:** Plasmids and glycerol stocks can be stored at -20**º**C until sequence has been confirmed.

**CRITICAL STEP:** In case of ligation-mediated sgRNA cloning, the last base pair next to the junction point is deleted in rare cases, so it is advisable to check 1-2 colonies for each sgRNA clone to ensure the appropriate sequence of the sgRNA.

*Generation of HR template (TIME: ~ 2.5 hrs)*

1. Prepare PCR mastermix as in table below.

|  | **final concentration** | **Volume to add for 1 reaction (50 μl)** |
| --- | --- | --- |
| HR primer mix (10 μM per primer) | 0.4 nM/primer | 2 μl |
| Phusion HF-buffer (2X) | 1x | 25 μl |
| H2O |  | 23 μl |

1. Perform PCR following protocol below.

| **Cycles** | **Temperature** | **Duration** |
| --- | --- | --- |
| 1 cycle | 98ºC | 2 min |
| 30 cycles | 98ºC | 10 sec |
|  | 55ºC | 10 sec |
|  | 72ºC | 30 sec |
| 1 cycle | 72ºC | 5 min |

1. Check PCR products by running 5 µl on 1.5 % agarose TBE gel.

**PAUSE POINT:** HR template can be stored at -20 **º**C.

*Preparation of synchronized competent cryopreserved S. pombe cells (TIME: ~1.5 days)*

*Preparation of synchronized competent cryopreserved S. pombe cells (TIME: ~1.5 days)*

This protocol is a modification of a previously described method [12] to prepare 200 ml of competent cells that allows for 40 transformations.

1. Prepare 20 ml preculture in EMM and grow cells by shaking at 32**º**C for 8-16 hrs.
2. Dilute cells in 200 ml EMM and grow cells until they reach mid-exponential phase (~2 x 10^9^ cells in total).
3. Centrifuge cells for 3 min at 1800g, room temperature.
4. Remove supernatant.
5. Wash in one volume (200 ml) of EMM without nitrogen (EMM-N).
6. Repeat steps 45 and 46 once more.
7. Resuspend cells in 200 ml of EMM-N and transfer to sterile flask.
8. Incubate for 2 hrs at 25**º**C with shaking.
9. Check that cells have become smaller and rounder, under light microscope.
10. Place cell culture on ice for 15 min.

**CRITICAL STEP:** To maintain integrity and competency of cells, they must be kept at 4ºC from this moment.

1. Centrifuge cells for 5 min at 1600g, 4**º**C and remove supernatant.
2. Resuspend cells on **ice-cold**, sterile water.
3. Centrifuge for 5 min at 1600g, 4**º**C and remove supernatant.
4. Repeat steps 54 and 55 twice more.
5. Resuspend cells in 2 ml of **ice-cold,** filter-sterilized 30%Glycerol, 0.1M Lithium acetate (pH 4.9), which gives 10^9^ cells/ml.
6. Prepare 50 µl cell aliquots in 1.5 ml sterile Eppendorf tubes, place aliquots on ice for 2 min. Each aliquot is for one transformation.
7. Store aliquots at -80**º**C immediately.

**PAUSE POINT:** Cryopreserved cells can be stored at -80**º**C for months.

*Transformation of cryopreserved S. pombe cells (TIME: 30 min)*

1. Thaw aliquots of synchronized, cryopreserved cells in dry block at 40**º**C for 2 min.
2. For each transformation, add 2µl of 10 µg/µl denaturated herring sperm DNA, 10 µl of HR template, and 2 µg of gRNA plasmid (~10 µl of standard mini-prep yield of 200ng/µl).
3. Add 145 µl of 50% PEG4000, mix well and immediately incubate mix for 15 min at 43**º**C.
4. Centrifuge cells for 3 min at 1600g, room temperature.
5. Remove supernatant and resuspend cells in 1 ml of EMM-N.

**CRITICAL STEP:** In the case of using auxotrophic mutant, we recommend the addition of 1/10 of usual concentration of relevant supplement. If using an *h^90^* strain, use EMM with nitrogen to prevent sporulation.

1. Incubate at room temperature for 16 hrs, without shaking.
2. Centrifuge cells for 3 min 1600g, room temperature.
3. Remove supernatant and plate all cells on YES plates containing 100 µg/ml of Noursethricin.
4. Incubate plates at 32**º**C for at least 4 days (longer incubations are sometimes required).
5. Re-streak smallest colonies onto YES plates. Cas9 expression is deleterious for cells, and re-streaking onto non-selective YES allows for elimination of the Cas9 plasmid.

**CRITICAL STEP:** It is important to select the smallest colonies present (Figure 5): Large colonies are likely to emerge from transformants with mutations or rearrangements of Cas9 [11], and this problem is compounded in the large and unstable Cas9-sgRNA plasmid. Unpublished data suggest that the mutations happen during *E. coli* growth (large colony counts fluctuate between miniprep cultures but are quite stable within one culture), and may get worse with freeze-thaws of the plasmid (more negative large colonies in freeze-thawed plasmids).

.

*Checking of deletions by colony PCR (TIME: 4 hrs)*

1. Prepare master mix for PCR reactions following table below:

| **Components** | **Final concentration** | **Volume per reaction (25 μl total)** |
| --- | --- | --- |
| Cell colony | -- | Scoop cells with tip of pipette |
| TopTaq PCR Buffer 10x | 1x | 2.5 µl |
| MgCl_2_ 25 mM | 0.5 mM | 0.5 µl |
| Primer mix (10 µM per primer) | 0.4 nM/ each primer | 1 µl |
| dNTPs mix (10 µmol per dNTP) | 0.4 nmol/ each | 1 µl |
| Q Solution 5x | 1x | 5 µl |
| H_2_O | - | 14.75 µl |
| TopTaq DNA Polymerase (5 U/ µl) |  | 0.25 µl |

1. For each colony take a little biomass with 10 µl pipette tip and resuspend in PCR mix (with this polymerase there is no need to boil cells prior to PCR).
2. Perform PCR following protocol below.

| **Cycles** | **Temperature** | **Duration** |
| --- | --- | --- |
| 1 cycle | 94ºC | 2 min |
| 35 cycles | 94ºC | 30 sec |
|  | 52ºC | 30 sec |
|  | 72ºC | 2min 30 sec |
| 1 cycle | 72ºC | 7 min |

1. Add 5 µl of loading dye and load 10 µl of PCR product with loading dye mix on 0.7% agarose gel. The sizes of expected products are indicated in the output of CRISPR4P. (For successful deletions, this will be ~200 bp and for wild-type, the size of target region to delete plus ~200 bp of flanking regions).

OPTIONAL: add 0.5 µl of 10 mg/ml RNAse A solution to mix before loading to remove RNA that might complicate band visualization on gel.

To confirm the deletion junction, the PCR products can be sent for Sanger sequencing (the expected sequence surrounding the deletion junction is provided by CRISPR4P). Figure 3F provides an example of an agarose gel showing successful deletions.
