# Supplementary material for: A CRISPR/Cas9-based method and primer design tool for seamless genome editing in fission yeast
Source: Wellcome Open Res. 2017 May 5;1:19. Originally published 2016 Nov 23. [Version 3] doi: 10.12688/wellcomeopenres.10038.3 (PMC5445975; doi:10.12688/wellcomeopenres.10038.3)
Supplement: Peer review history

# Peer review: Referee response for version 2

**DOI:** 10.21956/wellcomeopenres.11382.r19374

**Published:** 2017-01-16

**Authors:** Damien Hermand

**Affiliations:** 1 Namur Research College, The University of Namur, Namur, Belgium

Our comments were addressed with this revised version and I have no further comments.

I have read this submission. I believe that I have an appropriate level of expertise to confirm that it is of an acceptable scientific standard.

# Peer review: Referee response for version 2

**DOI:** 10.21956/wellcomeopenres.11382.r18893

**Published:** 2017-01-04

**Authors:** Silke Hauf

**Affiliations:** 1 Department of Biological Sciences and Biocomplexity Institute, Virginia Tech, Blacksburg, VA, USA

I have read this submission. I believe that I have an appropriate level of expertise to confirm that it is of an acceptable scientific standard.

# Peer review: Referee response for version 1

**DOI:** 10.21956/wellcomeopenres.10814.r17926

**Published:** 2016-12-05

**Authors:** Damien Hermand, Olivier Finet, Carlo Yague-Sanz

**Affiliations:** 1 Namur Research College, The University of Namur, Namur, Belgium

The implementation of the CRISPR/Cas9 system in fission yeast by the Zaratiegui laboratory created a large interest within the community and many laboratories have tried to set up the method with apparently low success. Therefore, the present work by the Zaratiegui and Bahler groups is important and will help to solve some issues. It is expected that the improvements presented here will spread within the community. A new selection is proposed for the Cas9 vector together with a new transformation method. A straightforward web tool is added to the already long list of very useful resources developed over the years by the Bahler lab.

 The manuscript is well written and most of the time easy to read. Solving the following issues will improve clarity.

- In Figure 2, in « Table », the « numbers on the right » may be explained with a simple sentence. Also, the naming « Table » seems a bit odd.
- The colour code used in Figures 3 and 4 is different for the sg primers, which may be misleading to some readers.
- In Figure 5, the red arrows do not seem to point to anything while supposed to highlight small colonies. Maybe using red circles will be better.
- Figure 6 is not easy to understand. According to Figure 6A, there are 29 deletions with a percentage of succesful deletions between 0 and 10% while the legend of Figure 6B suggests that 38 sgRNAs (36 sgRNAs #1 and 2 sgRNAs #2) did not yield to successful deletions. How is this possible? A table in the supplementary data may be easier to read if showing for every ncRNAs: the number of sgRNA tried and for each sgRNAs, the succes rate. The abscissa axis in Figure 6B is especially very hard to read.
- It would also be useful to discuss if the genome position matters and if the list of the targeted ncRNAs roughly covers the whole genome.
- The paragraph related to point mutations could provide more details or be removed and inserted later on when more data are available.

Other comments.

- It may be useful to mention that commercial kits are available to introduce the sgDNA into the vector, for example the BioLabs Q5® Site-Directed Mutagenesis Kit that is inexpensive and efficient.
- The code on GitHub should be at least minimaly documented.
- The version of Pombase used to build the database should be indicated.
- There are still quite a few typos and mistakes, for examples:
  on page 3 : The data to the right of each sgRNA indicates the numbers of other genomic sgRNA sequences that share a given number of nucleotides (starting from the 5’ end of the PAM sequence), isn’t « the number of genomic sequences » rather than the « the number of OTHER genomic sequences »?
  on page 6: rkk1-guided sgRNA should be rrk1-guided sgRNA

We have read this submission. We believe that we have an appropriate level of expertise to confirm that it is of an acceptable scientific standard, however we have significant reservations, as outlined above.

# Response

**Published:** 2016-12-21

**Authors:** Jürg Bahler

**Affiliations:** Department of Genetics, Evolution and Environment, University College London, UK

We thank the reviewers for their helpful and constructive comments. Below we provide a point-by-pint response to the specific issues raised (pasted in italic).

- *In Figure 2, in « Table », the « numbers on the right » may be explained with a simple sentence. Also, the naming « Table » seems a bit odd.*

We have changed the title of this Table to ‘Suggested sgRNAs’ in both the web tool and in Figure 2. In the figure legend, we have also provided an explanation for the numbers on the right of the table.

- *The colour code used in Figures 3 and 4 is different for the sg primers, which may be misleading to some readers.*

We have now modified Figure 4 to match the color of the sgRNA primers with the ones in Figure 3.

- *In Figure 5, the red arrows do not seem to point to anything while supposed to highlight small colonies. Maybe using red circles will be better.*

We want to highlight the very smallest colonies because these are the most likely to be correct. In the screen version of the figure, these colonies are visible. We have increased the contrast to better visualize these colonies and now highlight small colonies using red circles as suggested.

- *Figure 6 is not easy to understand. According to Figure 6A, there are 29 deletions with a percentage of succesful deletions between 0 and 10% while the legend of Figure 6B suggests that 38 sgRNAs (36 sgRNAs #1 and 2 sgRNAs #2) did not yield to successful deletions. How is this possible? A table in the supplementary data may be easier to read if showing for every ncRNAs: the number of sgRNA tried and for each sgRNAs, the succes rate. The abscissa axis in Figure 6B is especially very hard to read.*

We understand that this was confusing. We have now corrected the x-axis of Figure 6A to indicate that the lowest bin contains 1-10% of successful deletions (instead of 0-10%). We have also clarified this in the figure legend. Here we only show the percentages of successful deletions. We did not add numbers for any unsuccessful deletions, because we cannot be certain whether they failed because of the sgRNA sequence, mutations in the plasmid, or any other reason. We now also provide a Supplementary Table 2 showing the data from Figures 6A and 6B, which is cited in the legend of Figure 6.

- *It would also be useful to discuss if the genome position matters and if the list of the targeted ncRNAs roughly covers the whole genome*

We have deleted ncRNA genes spread across all 3 chromosomes. We have now added an additional section C to Figure 6 to show the genomic positions of all annotated ncRNA genes (grey dots) and the ncRNA genes that we have successfully deleted (red dots). As for all genome manipulations, there may of course be genomic regions which are less amenable to changes, e.g. due to inaccessible chromatin.

- *The paragraph related to point mutations could provide more details or be removed and inserted later on when more data are available.*

We think that it is helpful to report at this point that it is also possible to get point mutations using our CRISPR/Cas9 method. We will expand on this in a future update of the paper.

- *It may be useful to mention that commercial kits are available to introduce the sgDNA into the vector, for example the BioLabs Q5® Site-Directed Mutagenesis Kit that is inexpensive and efficient.*

We know this kits, but have optimized the conditions for the polymerase indicated. A different polymerase may require optimization of the initial PCR reaction, and the specific protocol provided may no longer be valid in all details. Naturally, other users can experiment with different procedures or kits, and we would be interested to hear of any alternatives that have been implemented.

- *The code on GitHub should be at least minimaly documented.*

We now provide basic documentation of the code in GitHub.

 The version of Pombase used to build the database should be indicated.

 The genome assembly is ASM294v2 and the annotation version 55, which is now specified in the text.

- *There are still quite a few typos and mistakes, for examples:*
**
* on page 3 : The data to the right of each sgRNA indicates the numbers of other genomic sgRNA sequences that share a given number of nucleotides (starting from the 5’ end of the PAM sequence), isn’t « the number of genomic sequences » rather than the « the number of OTHER genomic sequences »?*
- *on page 6: rkk1-guided sgRNA should be rrk1-guided sgRNA*

We have corrected these typos as suggested.

# Peer review: Referee response for version 1

**DOI:** 10.21956/wellcomeopenres.10814.r17928

**Published:** 2016-12-05

**Authors:** Silke Hauf

**Affiliations:** 1 Department of Biological Sciences and Biocomplexity Institute, Virginia Tech, Blacksburg, VA, USA

Rodriguez-Lopez and colleagues report improvements on CRISPR/Cas9-mediated genome editing in *S. pombe* (fission yeast). Their work builds on a previous paper by Jacobs, Zaratiegui *et al.* (2014). In this earlier paper, Jacobs *et al.* describe an expression vector for Cas9 and sgRNA that functions in *S. pombe* and demonstrate that mutagenesis and epitope-tagging is possible with this system.

 Rodriguez-Lopez *et al.* now make several changes to improve the efficiency and the ease of use.

1. The authors change the auxotrophic selection marker *ura4*+ in the Cas9/sgRNA plasmid to a nourseothricin-resistance, which does not require a specific genetic background and allows selection on rich medium.
  Another group has recently implemented a fluoride export channel as another marker replacing *ura4*+ on this plasmid, which also allows selection on rich medium and accelerates Cas9-mediated genome editing (Fernandez and Berro 2016). This also works well in our hands. I think it would be great if the authors cited this paper, so that readers are aware of all the different possibilities.
  Non-specific double-strand breaks created by Cas9 are always a concern, and it is important that the plasmid is efficiently lost after successful genome modification. With the *ura4*+ version, 5-FOA could be used for counterselection. Since expression of Cas9 impairs growth, I am assuming that loss of the plasmid is very efficient, even without counterselection. However, if the authors happen to have data on this, it would be nice to mention it. (For example, how many clones lost the plasmid after one passage on non-selective medium?)
2. The authors have written a program to select specific sgRNA target regions. The program then suggests primers for sgRNA cloning, as well as primers to delete a gene of interest and to check for successful deletion.
  This is generally very useful. When I tried the online version, I had no problem specifying a gene by name, but finding sgRNA targets by entering specific coordinates did not work for me (using two different browsers). My input caused an "Internal Server Error". It would be great if the authors could look into this.
  The current implementation of the program suggests sgRNA targets based on specificity, but - as the authors show - efficiency can be highly variable. For *S. pombe*, there is no data available to indicate which target regions may be particularly efficient (and even in other organisms, information is still scarce). I was wondering whether it could be useful to extend the program to allow community feedback (i.e. when a researcher is using one of the suggested sgRNAs, she/he could input how well this site worked). This would (a) avoid that several people try using target regions that are not efficient, and (b) in the long run maybe allow it to determine which factors influence efficiency. Obviously, some sort of quality control on the user input would be required (e.g. number of successful genome modifications per how many clones tested, and a gel picture to support this), which may make it too time-consuming for the Bähler Lab to curate.
3. The previous system needed digest of the Cas9/sgRNA plasmid with the restriction enzyme *Csp*CI, which sometimes is inefficient. The authors have now solved this problem by amplification of the entire 11 kb plasmid with primers that contain the specificity region, followed by ligation. This seems more inconvenient than is necessary. However, the authors already mention that they work on other improved strategies for the sgRNA cloning step that they will add to the paper as they are implemented. I agree that this will be highly useful. 
4. The authors have further improved an existing protocol for *S. pombe* transformation to increase transformation and deletion efficiency. Figure 5 shows that G1 synchronization greatly improves transformation efficiency. If the authors happen to have data to which extent the cryopreservation affects this result, it would be great if they could add it.

 

 Overall, these are all highly useful changes - a clear improvement over the previous protocol. Motivation and results are well described, and the step-by-step procedure is detailed and easy to follow.

I have read this submission. I believe that I have an appropriate level of expertise to confirm that it is of an acceptable scientific standard.

## References

[1] JacobsJZCiccaglioneKMTournierVZaratieguiM: Implementation of the CRISPR-Cas9 system in fission yeast. *Nat Commun*.2014;5: 10.1038/ncomms6344 5344 10.1038/ncomms6344 25352017PMC4215166

[2] FernandezRBerroJ: Use of a fluoride channel as a new selection marker for fission yeast plasmids and application to fast genome editing with CRISPR/Cas9. *Yeast*.2016;33(10) : 10.1002/yea.3178 549-557 10.1002/yea.3178 27327046PMC5363073

# Response

**Published:** 2016-12-21

**Authors:** Jürg Bahler

**Affiliations:** Department of Genetics, Evolution and Environment, University College London, UK

We thank the reviewer for her helpful, constructive comments. Our response to the specific issues raised (pasted in italic) is presented below.

- *The authors change the auxotrophic selection marker ura4+ in the Cas9/sgRNA plasmid to a nourseothricin-resistance, which does not require a specific genetic background and allows selection on rich medium.*
* Another group has recently implemented a fluoride export channel as another marker replacing ura4+ on this plasmid, which also allows selection on rich medium and accelerates Cas9-mediated genome editing (Fernandez and Berro, 2016). This also works well in our hands. I think it would be great if the authors cited this paper, so that readers are aware of all the different possibilities.*

We missed this paper, thank you. We have now cited it in the introduction. A drawback of is that fluoride selection requires a specific strain background.

- *Non-specific double-strand breaks created by Cas9 are always a concern, and it is important that the plasmid is efficiently lost after successful genome modification. With the ura4+ version, 5-FOA could be used for counterselection. Since expression of Cas9 impairs growth, I am assuming that loss of the plasmid is very efficient, even without counterselection. However, if the authors happen to have data on this, it would be nice to mention it. (For example, how many clones lost the plasmid after one passage on non-selective medium?)*

We have checked 106 colonies after one pass onto non-selective media, and 88 (83%) of these colonies have lost the plasmid passively.

- *The authors have written a program to select specific sgRNA target regions. The program then suggests primers for sgRNA cloning, as well as primers to delete a gene of interest and to check for successful deletion.This is generally very useful. When I tried the online version, I had no problem specifying a gene by name, but finding sgRNA targets by entering specific coordinates did not work for me (using two different browsers). My input caused an "Internal Server Error". It would be great if the authors could look into this.*

This bug has been corrected.

- *The current implementation of the program suggests sgRNA targets based on specificity, but - as the authors show - efficiency can be highly variable. For S. pombe, there is no data available to indicate which target regions may be particularly efficient (and even in other organisms, information is still scarce). I was wondering whether it could be useful to extend the program to allow community feedback (i.e. when a researcher is using one of the suggested sgRNAs, she/he could input how well this site worked). This would (a) avoid that several people try using target regions that are not efficient, and (b) in the long run maybe allow it to determine which factors influence efficiency. Obviously, some sort of quality control on the user input would be required (e.g. number of successful genome modifications per how many clones tested, and a gel picture to support this), which may make it too time-consuming for the Bähler Lab to curate.*

Yes, we agree this is a good idea. As already mentioned in the paper, we are assembling a database with all sgRNAs used, whether they worked or not, to help with learning the principles for successful sgRNAs in *S. pombe*. We may implement such a community system in a future update of the paper.

- *The previous system needed digest of the Cas9/sgRNA plasmid with the restriction enzyme CspCI, which sometimes is inefficient. The authors have now solved this problem by amplification of the entire 11 kb plasmid with primers that contain the specificity region, followed by ligation. This seems more inconvenient than is necessary. However, the authors already mention that they work on other improved strategies for the sgRNA cloning step that they will add to the paper as they are implemented. I agree that this will be highly useful.* 

We are working on a method to avoid the PCR cloning, and once this is implemented will report it in a future update of the paper.  

- *The authors have further improved an existing protocol for S. pombe transformation to increase transformation and deletion efficiency. Figure 5 shows that G1 synchronization greatly improves transformation efficiency. If the authors happen to have data to which extent the cryopreservation affects this result, it would be great if they could add it.*

We have not systematically checked to what extent cryopreservation helps, but it improves the procedure in our hands. In the original paper describing the protocol, Suga et al. report that the solution containing glycerol used as cryoprotectant improves efficiency: *“T* *hese permeating agents have an ability not only to cryoprotect cells but also to improve transformation efficiency, and glycerol was a more effective agent for Sz. pombe cells. Thus, the thawed competent cells could be used directly for transformation without removing the glycerol because the presence of glycerol in the transformation mixture was important.”*

 But the G1 synchronization seems to make an even a bigger difference, and we have further specified this in the legend of Figure 5. Transformation of synchronous cells consistently resulted in 3-fold to over 1000-fold higher numbers of colonies than transformation of asynchronous cells.
